# GA_4_/GA_7_ Deficiency and Downregulated Ent-Kaurenoic Acid Oxidase Impair Seedless Mango Fruit Development

**DOI:** 10.3390/foods14213705

**Published:** 2025-10-30

**Authors:** Meng Gao, Songbiao Wang, Wentian Xu, Wenxin Li, Xiaowei Ma

**Affiliations:** 1 Key Laboratory of Tropical Fruit Biology, Ministry of Agriculture & Rural Affairs/Key Laboratory of Hainan Province for Postharvest Physiology and Technology of Tropical Horticultural Products, South Subtropical Crops Research Institute, Chinese Academy of Tropical Agricultural Sciences, Zhanjiang 524091, China; nysgm@catas.cn (M.G.); wangsongbiao@catas.cn (S.W.); xuwentian@catas.cn (W.X.); 2 Sanya Research Institute, Chinese Academy of Tropical Agricultural Sciences, Sanya 572024, China

**Keywords:** mango, seedless fruit, gibberellins, fruit development, KAO

## Abstract

Seedless mangoes are desirable for fresh consumption and processing; however, they frequently exhibit poor fruit development and elevated abscission rates, necessitating applications of fruit regulators, though their physiological and molecular targets remain unclear. In this study, hormonal deficits and regulatory genes associated with mango fruit development were identified. Morphological observations showed that embryo-containing (EC) and embryo-absent (EA) fruits begin to diverge in development between 30 and 45 days after bloom (DAB). During this period, targeted metabolomics of gibberellins (GAs) detected nine GAs; four (GA_20_, GA_34_, GA_4_, GA_7_) behaved consistently across cultivars, with GA_4_ and GA_7_ showing the largest declines in EA fruit. Applying 50 mg/L GA_3_ or GA_4+7_ at 30 DAB increased fruit growth versus water, with GA_4+7_ having the greatest effect. Comparative transcriptomic analysis revealed 1476 shared DEGs that were enriched in hormone signaling pathways. Among 13 DEGs involved in GA biosynthesis, *KAO* (*Mi05g23760.1*) showed the strongest correlation with GA levels and was markedly downregulated in EA fruits. Together, these results indicate that deficiencies in GA_4_/GA_7_—potentially resulting from reduced KAO expression—contribute to impaired EA fruit development and that targeted GA_4+7_ supplementation may more effectively rescue growth than conventional GA_3_ treatments. These results provide mechanistic insights and practical guidance for hormone-based strategies to promote consistent development of seedless mangoes, thereby improving yield stability and supporting more sustainable production.

## 1. Introduction

Mangoes (*Mangifera indica* L.) are among the most widely consumed and cherished fruits worldwide, celebrated for their sweet, juicy pulp and vibrant colors. As a cornerstone of tropical fruit agriculture, mangoes also serve as a vital raw material for the food processing industry, providing abundant supply for products such as juices, purees, dried snacks, and functional food ingredients [1,2]. The presence of large seeds in the central cavity of fruits is often considered a drawback, as they impede direct consumption and complicate industrial processing [3]. Seedless fruits are increasingly favored in agricultural production for their higher edible yield and greater suitability for food processing; in mangoes, inadequate pollination often induces seedlessness, enhancing commercial appeal [4,5]. Nonetheless, growing seedless mangoes presents challenges including reduced yield and quality, high rates of fruit drop, poor development, and food safety concerns from the irrational use of growth regulators [6]. Advancing our understanding of the biological and physiological mechanisms driving seedless mango development could improve fruit quality traits (e.g., texture, sugar-acid balance, and shelf stability), enabling better processing adaptability and nutritional retention for food applications.

The development of mango fruit begins at the flowering stage and is influenced by cultivar and climatic conditions, with maturation typically requiring 120 to 160 days [7]. Throughout this period, notable alterations in the size, color, and biochemical composition of the fruits take place, which are crucial determinants of their quality [8]. Although seedless mangoes enhance taste quality and possess superior overall flavor, they are highly susceptible to improper development and deformities [9,10]. To meet the demands of high-quality fruit production without the natural occurrence of seeds, seedless fruit development relies heavily on the application of exogenous phytohormones, particularly CPPU (N-(2-chloro-4-pyridyl)-N′-phenylurea) and gibberellins (GAs) [11]. CPPU, a synthetic cytokinin, has found widespread use predominantly because of its cost-effectiveness and reliable results in improving yield and quality compared to natural fruits. A 20 mg/L CPPU treatment applied to ‘Hongyang’ kiwifruit 20 days after pollination increased fruit size and mass and improved overall fruit quality by elevating soluble sugar and ascorbic acid concentrations and promoting cell expansion and division [12]. The role of gibberellins is equally crucial, as they directly interact with cytokinin pathways to fulfill the developmental processes. Application of GA_3_ (20 ppm) and the synthetic cytokinin CPPU (5 ppm), either singularly or in combination, to ‘Sable Seedless’ grape fruitlets resulted in increased berry size, and concomitantly delayed sugar accumulation and acid degradation, with effects being more pronounced for CPPU [13]. The quantity of seeds within a berry influences its size, while the absence of seeds can partially be compensated by exogenous gibberellin application [14]. Although CPPU and GA_3_ have been extensively researched in the context of seedless fruit development, there have been few reports on the primary hormonal differences between seeded and seedless fruits.

GAs are pivotal plant hormones that influence various growth processes, including seed germination, stem elongation, and fruit development [15]. Notable gibberellins such as GA_1_, GA_3_, GA_4_, and GA_7_ are integral to these processes [16]. In rice, GA_1_ predominantly affects vegetative growth, whereas GA_4_ enhances the interaction with the gibberellin receptor GID1, facilitating the degradation of DELLA proteins which typically inhibit gibberellin signaling [17,18]. In spinach, exposure to far-red-enriched long-day conditions stimulates stem elongation and flowering, processes that depend on gibberellin synthesis; these can be altered by inhibitors and reversed through GA_3_ treatment [19]. Additionally, the combination of GA_4_ and GA_7_ is critical for inducing pseudo-embryo formation and parthenocarpy in pear, regulated by the PbDELLA-PbMYB56-PbCYP78A6 module [20]. The biosynthesis of bioactive GAs begins with the conversion of geranylgeranyl diphosphate (GGDP) into the intermediate ent-kaurene by the terpene synthases ent-copalyl diphosphate synthase (CPS) and ent-kaurene synthase (KS) in the plastids. Subsequently, ent-kaurene is oxidized by *ent-kaurene oxidase* (*KO*) and *ent-kaurenoic acid oxidase* (*KAO*), both cytochrome P450 monooxygenases (P450s), leading to the formation of GA_12_ [21]. Following the production of GA_12_, *GA_20_-oxidase* (*GA20ox*) and *GA_3_-oxidase* (*GA3ox*), are involved in the synthesis of bioactive GAs, such as GA_4_ and GA_7_. Additionally, the enzyme *GA_13_-oxidase* (*GA13ox*) catalyzes the conversion of GA_12_ to GA_53_, further facilitating the synthesis of GA_1_ and GA_3_. GA_3_ is the primary growth regulator used in seedless mango production; however, optimizing its application requires a precise understanding of endogenous gibberellin deficiencies in seedless cultivars. Identifying the specific gibberellins that are deficient would enable development of more targeted and effective hormonal treatment protocols.

In this study, an in-depth analysis of gibberellin levels in both seeded and seedless ‘Keitt’ and ‘Jinbaihua’ mangoes was performed during crucial developmental stages. Our results indicate that despite the widespread commercial application of gibberellin GA_3_, the principal shortcomings in seedless mangoes are primarily associated with gibberellins GA_4_ and GA_7_. Further investigations highlighted the key role of the KAO gene in mediating these hormonal disparities. This study elucidates the hormonal regulation mechanisms underlying the development of seedless mango fruits, offering valuable insights for optimizing postharvest processing and quality preservation in food manufacturing. The findings present potential applications for developing hormone-based strategies to enhance the texture, shelf life, and nutritional properties of processed mango products, thereby contributing to advancements in food science and technology.

## 2. Materials and Methods

### 2.1. Plant Materials

Embryo-containing (EC) and embryo-absent (EA) fruits of ‘Keitt’ and ‘Jinbaihua’ mango cultivars were collected at 30 and 45 days after bloom (DAB), while EA ‘Jinhuang’ fruits were sampled at 45, 60, and 75 DAB based on previous study [19]. All samples were obtained from the South Subtropical Crops Research Institute (SSCRI) orchard (21°12′ N, 110°4′ E) in Mazhang County, Guangdong, China. Fruit vertical and transverse diameters were measured using digital vernier calipers. Three biological replicates pooled from six fruits were used. Fruit index was calculated as the ratio of fruit vertical diameter to fruit transverse diameter. The pulp was manually separated from the fruit samples, immediately diced, frozen in liquid nitrogen, and stored at −80 °C for gibberellin (GA) content and RNA-Seq analysis. Each developmental stage was sampled with three biological replicates, with each replicate consisting of pooled flesh from six fruits.

### 2.2. Detection of Gibberellins

GA contents were analyzed by MetWare (http://www.metware.cn/, accessed on 26 May 2025) as an analytical service using an AB Sciex QTRAP^®^ 6500+ LC–MS/MS system. Fresh plant samples were flash-frozen in liquid nitrogen, ground into powder, and extracted with methanol/water/formic acid (15:4:1, *v*/*v*/*v*). After liquid–liquid partitioning with ethyl acetate, the extract was derivatized using BPTAB/TEA (90 °C, 1 h) and reconstituted in ACN/water (90:10, *v*/*v*). Chromatographic separation was performed on a Waters CSH column (2.1 × 100 mm, 1.7 μm) with a gradient of 0.05% formic acid in water (A) and acetonitrile (B) (5–95% B over 14 min) at 0.35 mL/min. Mass spectrometry detection was conducted in positive ion mode (IS 5500 V) using multiple reaction monitoring, with optimized parameters including curtain gas (35 psi), ion source temperature (550 °C), collision energy and declustering potential shown in Table S1. Data acquisition and metabolite quantification were performed using Analyst 1.6.3 and Multiquant 3.0.3 software, respectively. The results of hierarchical cluster analysis (HCA) for samples and metabolites were visualized as heatmaps with dendrograms. HCA was performed using the R package (version 4.5.1) pheatmap. Normalized metabolite signal intensities (unit variance scaling) were represented using a color spectrum. Significantly regulated metabolites between groups were determined by absolute log2 fold change ≥ 1.

### 2.3. GA_3_ and GA_4+7_ Treatment

Ten uniformly growing ‘Jinhuang’ mango trees were selected. A 50 mg/L GA_3_ solution was sprayed once at both the early flowering and full bloom stages to induce EA fruit formation (induction rate > 90%). GA_3_ was first dissolved in absolute ethanol to make a 10 g/L stock solution, and this stock was diluted with deionized water to a final concentration of 50 mg/L immediately before application. At 15 DAB, three treatments were applied: 50 mg/L GA_3_, 50 mg/L GA_4+7_, and water (control), with 30 fruiting shoots per treatment. Before treatment, fruit thinning was conducted, retaining approximately 10 uniformly sized fruits per shoot. Fruit clusters were immersed in the solution for 5 s. Treatments were performed at 25 ± 2 °C under a 16 h light/8 h dark photoperiod and ~60–70% relative humidity; plants/fruit clusters were held under these conditions for the duration of the experiment unless otherwise stated. Because published studies of exogenous GA application in mango are limited, we based our sampling schedule on well-documented GA studies in grapevine, which report that the largest fruit developmental responses occur at approximately 30 days after GA application [13]. Accordingly, sampling in this study began 30 days after GA treatment, corresponding to 45, 60 and 75 DAB.

### 2.4. Transcriptome Sequencing and Analysis

The cDNA libraries were sequenced on the Illumina sequencing platform by Metware Biotechnology Co., Ltd. (Wuhan, China). Total RNA was extracted from plant samples using a combined CTAB/PBIOZOL protocol; RNA was dissolved in DEPC-treated water, quantified with a Qubit fluorometer and assessed for integrity (RQN) on a Qsep400 biofragment analyzer. mRNA was enriched by oligo(dT) magnetic beads, fragmented, and reverse-transcribed to first-strand cDNA with random hexamers; second-strand synthesis incorporated dUTP for strand specificity while end repair and A-tailing were performed. Adapters were ligated, libraries were size-selected to ~250–350 bp inserts, PCR-amplified, purified, and quality-checked (concentration by Qubit; fragment size by Qsep400). Finally, libraries were pooled and sequenced on an Illumina platform as 150 bp paired-end reads. The raw sequencing data were processed using fastp to remove adapter sequences, reads with >10% N bases, and Q < 20. Clean reads were then aligned to the reference genome (PRJNA487154, NCBI) using HISAT [6]. RNA-seq data quality control and trimming were performed with FastQC, alignments were done with HISAT2, and differential expression analysis with DESeq2. Significantly differentially expressed genes were identified based on adjusted *p*-values and log2 fold change thresholds. Enrichment analysis was performed based on the hypergeometric test, with pathway-based hypergeometric distribution testing for KEGG and GO term-based analysis for GO. Raw transcriptomic data are deposited in the NCBI Sequence Read Archive under project ID PRJNA1347941.

### 2.5. Tissue-Specific Expression Patterns and Heatmap Visualization

The KAO gene expression profiles across different tissues were extracted from the transcriptomes of ‘Alphonso’ [6]. Expression values represent the mean of three biological replicates and were normalized by row using Z-scores for visualization. A heatmap was generated using the pheatmap package in R (v4.0.3), with log2-transformed and Z-score-normalized expression values to emphasize tissue-specific patterns. Hierarchical clustering was performed to group tissues with similar expression profiles, and a color gradient (red for high expression, blue for low expression) was applied for visualization.

### 2.6. Phylogenetic Tree Construction and Protein Alignment

A phylogenetic tree was constructed according to our previous research [22]. Amino acid sequences of Arabidopsis KAOs (AtKAO1, AT1G05160; AtKAO2, AT2G32440) were downloaded from TAIR (https://www.arabidopsis.org/, accessed on 26 May 2025). These protein sequences were used as queries to identify homologous genes in the mango reference genome: BLAST (version 2.17.0) searches were performed using NCBI BLAST+ (BLASTP against the predicted mango proteome, or TBLASTN against the genome assembly where necessary) with an E-value cutoff of 1 × 10^−5^ and identity/coverage filters. A phylogenetic tree was constructed according to the neighbor-joining method using previously described parameters of the MEGA (version X) program (https://www.megasoftware.net/, accessed on 26 May 2025). Evolutionary tree and multiple sequence alignment analyses were performed using Hiplot Pro (https://hiplot.com.cn/, accessed on 26 May 2025).

### 2.7. GAs and Their Metabolite Genes Correlation Analysis

To examine the relationships between GAs and their metabolic genes, we conducted Pearson correlation analysis using Hiplot Pro (https://hiplot.com.cn/, accessed on 26 May 2025). Gene expression levels and GA concentrations were log-transformed (if skewed) and normalized before analysis. Pairwise Pearson correlation coefficients (r) were computed to quantify linear dependencies, with statistical significance defined as *p* < 0.05 (FDR-adjusted). Results were visualized as a clustered heatmap, where color gradients (red: r > 0; blue: r < 0) indicated directional associations.

### 2.8. Statistical Analysis

All statistical analyses were performed using SPSS Statistics 27.0 (SPSS Inc., Chicago, IL, USA). The 2-way ANOVA and Student’s t test were used to evaluate the significance of any differences (* *p* ≤ 0.05, ** *p* ≤ 0.01, *** *p* ≤ 0.001). Bar graphs and line charts were generated using GraphPad Prism IX (GraphPad Software, San Diego, CA, USA).

## 3. Results

### 3.1. The Period 30 to 45 Days After Bloom Is Crucial for Seedless Fruit Development

**Figure 1:**
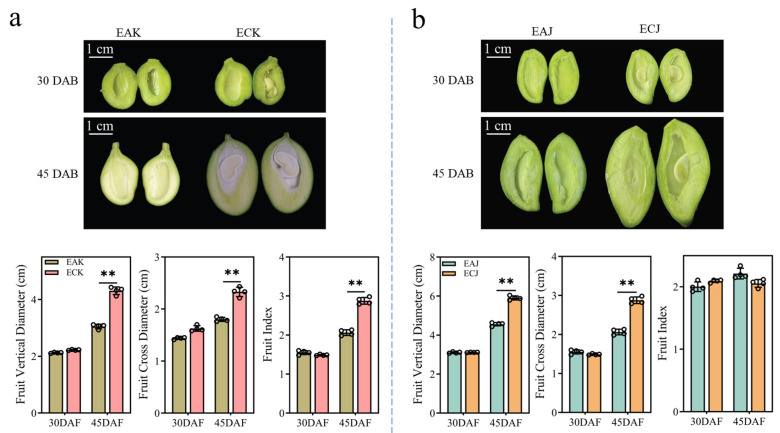
Influence of embryo presence on fruit dimensions and index in ‘Keitt’ and ‘Jinbaihua’. Images of ‘Keitt’ (**a**) and ‘Jinbaihua’ (**b**) fruits, both with and without embryos, are presented alongside measurements of their vertical and transverse diameters and corresponding fruit indices. Values represent mean ± SE (n = 3). Data were examined using two-way ANOVA, with asterisks indicating significant differences (** *p* < 0.01). Scale bars, 1 cm. EAK, embryo-absent ‘Keitt’ fruit; ECK, embryo-containing ‘Keitt’ fruit; EAJ, embryo-absent ‘Jinbaihua’ fruit; ECJ, embryo-containing ‘Jinbaihua’ fruit; DAB, days after blooming.

To delineate morphological differences between embryo-containing (EC) and embryo-absent (EA) mango fruits, we harvested early-stage EC and EA fruits of ‘Jinbaihua’ (elongated fruit type) and ‘Keitt’ (round fruit type) at 30 and 45 days after bloom (DAB). (Figure 1). Morphological evaluations at 30 DAB showed no significant differences between the EC and EA mango fruits. Nevertheless, at 45 DAB, both the vertical and horizontal diameters of the EA fruits were significantly reduced compared to EC fruits (Figure 1). The fruit shape index (vertical diameter/horizontal diameter) for EA ‘Keitt’ was significantly lower than that of its normal counterpart, suggesting that the elongation growth in EA fruits was markedly hindered (Figure 1a). Additionally, soluble solids content was approximately 1.2-fold higher in EA ‘Keitt’ fruits than in EC fruits, indicating superior quality of the EA fruits (Figure S2). These results indicate that the 30–45 DAB window is critical, as developmental anomalies in EA fruits typically emerge during this period. Therefore, we selected samples collected at 30 DAB for subsequent analysis of gibberellin (GA) concentrations and transcriptomic profiling.

### 3.2. Targeted Metabolomics of GAs Highlight GA_4_ and GA_7_ as Key Drivers of Mango Fruit Development

**Figure 2:**
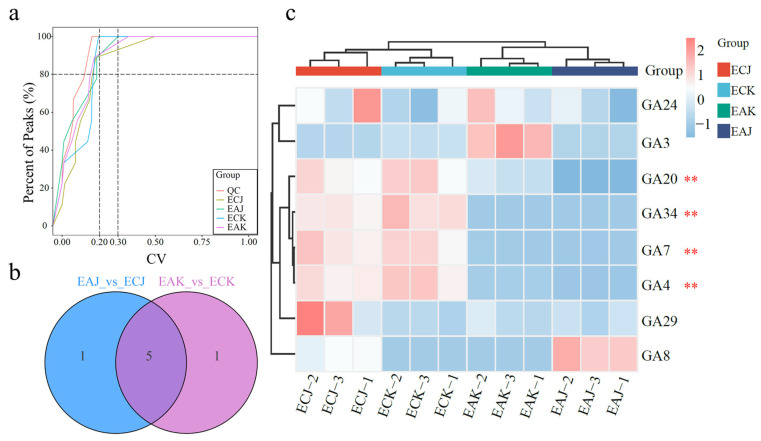
Endogenous gibberellin (GA) contents in EA and MA mango fruits at different developmental stages. (**a**) Distribution graph of coefficient of variation (CV) in each group of samples. Colors distinguish group samples, with QC for quality control. Lines at CV 0.2 and 0.3 and another line showing 80% of substances. (**b**) Venn diagram of differences among groups. (**c**) Overall clustering diagram of the samples. Horizontally represents the sample names, vertically represents the metabolite information, and different colors represent different values obtained after normalization of different contents (pink represents high content, green represents low content). Data were examined using two-way ANOVA, with asterisks indicating significant differences (** *p* < 0.01). GA content (ng g^−1^ FW).

GAs are plant hormones that promote growth and accelerate development while increasing fruit yield and quality. To analyze the effects of GAs on the development of fruits with different seed-bearing types, the targeted metabolomics of GAs within both EA and EC fruits of ‘Keitt’ and ‘Jinbaihua’ was measured, respectively. The coefficient of variation (CV) analysis indicates that substances with a CV value of less than 0.2 account for more than 80% of the quality control samples, demonstrating that the experimental data are very stable (Figure 2a). The Venn diagram illustrates the differences in metabolites between the ‘Keitt’ and ‘Jinbaihua’ cultivars, each containing one unique metabolite (Figure 2b). The intersection of the two sets represents five GAs common to both datasets (Figure 2b). Additionally, nine GAs were detected in ‘Keitt’ and ‘Jinbaihua’; five (GA_3_, GA_20_, GA_34_, GA_4_, GA_7_) differed between EA and EC fruits, but only GA_20_, GA_34_, GA_4_ and GA_7_ showed the same pattern in both cultivars (Table S2). Notably, GA_4_ and GA_7_ showed the most pronounced differences (Figure 2c), suggesting their deficiency as a potential critical factor hindering the development of non-embryonic fruits.

### 3.3. GA_4+7_ Outperforms GA_3_ in Promoting Development of EA ‘Jinhuang’ Mangoes

**Figure 3:**
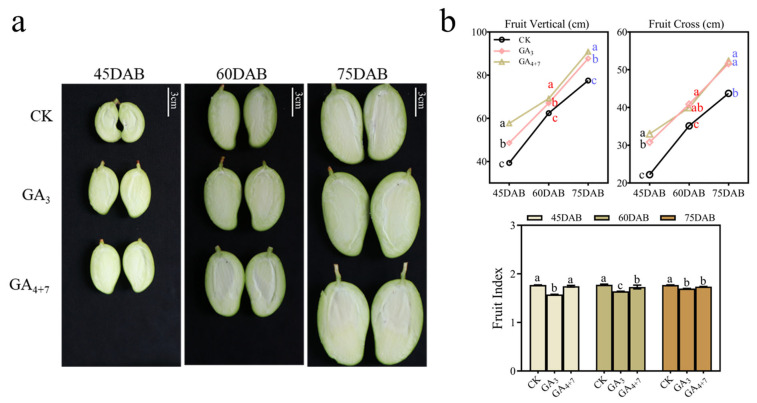
GA3 and GA4+7 treatments on seedless ‘Jinhuang’ mangoes. Images (**a**) and fruit morphological indices (**b**) of seedless ‘Jinhuang’ mangoes treated with GA_3_ and GA_4+7_. Data were analyzed using two-way ANOVA, different letters indicate significant differences (*p* < 0.05), scale bar, 3 cm. CK, control check; DAB, days after blooming.

In mango production, GA_3_ is commonly used as the primary regulator of fruit development. Our hormone metabolism analysis revealed that GA_4_ and GA_7_ are the most deficient gibberellins in EA mangoes. To preliminarily assess the effects of GA_4_ and GA_7_ on seedless fruit development, EA ‘Jinhuang’ mangoes at 30 DAB were treated with 50 mg/L GA_3_ or a combination of GA_4_ and GA_7_ (GA_4+7_), while the control group received water (Figure 3a). The results demonstrated that both GA_3_ and GA_4+7_ treatments significantly accelerated fruit development compared to the control, with GA_4+7_ exhibiting superior effects, especially promoting longitudinal growth (Figure 3b). These findings suggest that GA_4_ and GA_7_ regulate EA mango development more effectively than GA_3_.

### 3.4. Differential Transcriptomes of EA and EC Fruits Reveal Enrichment in Hormone Signaling and Secondary Metabolite Biosynthesis

**Figure 4:**
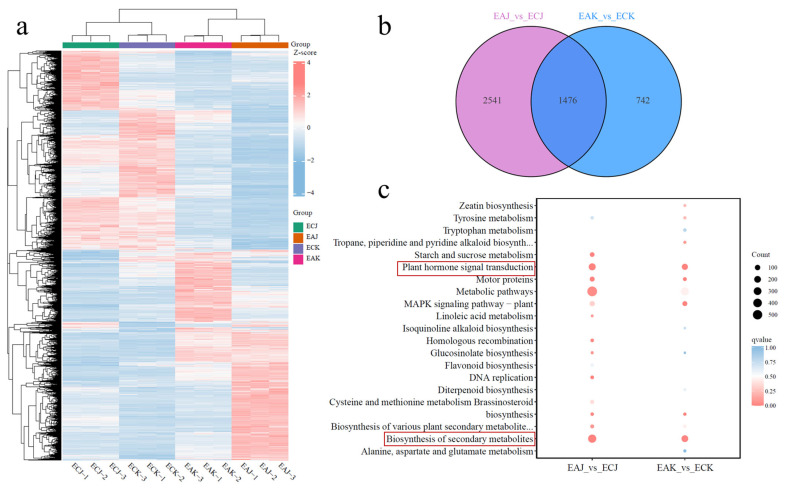
Embryo-absent and embryo-containing fruit transcriptomes. (**a**) Heatmap of hierarchical clustering of differentially expressed genes. the x-axis lists samples and their cluster results, while the y-axis features genes and their clustering. Pink indicates high expression; blue signifies low expression. (**b**) Venn diagram of differentially expressed genes among various groups. The non-overlapping area represents the differential genes unique to that differential group, while the overlapping area represents the differential genes shared by several overlapping differential groups. (**c**) Multiple combination KEGG enrichment scatter plot. The dot size shows how many differential genes are enriched in the pathway; bigger dots mean more genes. The dot color shows the enrichment significance; pink dots mean higher significance. The red boxes highlight the two key enriched KEGG pathways.

The gene expression differences of EA and with embryo EC fruits was assessed by comparing the transcriptome profiles. To identify genes differentially expressed between EA and EC fruits, we retained only those with a false discovery rate ≤ 5% and a ≥2-fold change in expression for analyses (Figure S2). After Z-score normalization and hierarchical clustering, heatmaps were generated for both the union of differentially expressed genes (DEGs) and each individual comparison group (Figure 4a). Hierarchical clustering and PCA analysis revealed that the two cultivars clustered closely within each fruit type (EC and EA), indicating similar gene-expression profiles within the same fruit type (Figure 4a and Figure S3a). By contrast, EA and EC fruits exhibited markedly different gene expression profiles (Figure 4a and Figure S3b). In total, we identified 4759 DEGs when comparing EA and EC fruits within the cultivars ‘Keitt’ and ‘Jinbaihua’ (Table S3). Of these, 1476 were shared between the two cultivars (Figure 4b). To better interpret the transcriptomic data, we also conducted a comparative KEGG pathway enrichment analysis of DEGs identified in EA and EC fruit. DEGs between EA and EC fruits were primarily enriched in plant hormone signal transduction and secondary metabolite biosynthesis (Figure 4c). These results indicate that embryo status substantially reprograms hormonal and metabolic gene networks, which are likely responsible for the observed differences in physiology and fruit quality between EA and EC fruits.

### 3.5. KAO Expression Associates with Divergent GA Profiles in EA and EC Fruits

**Figure 5:**
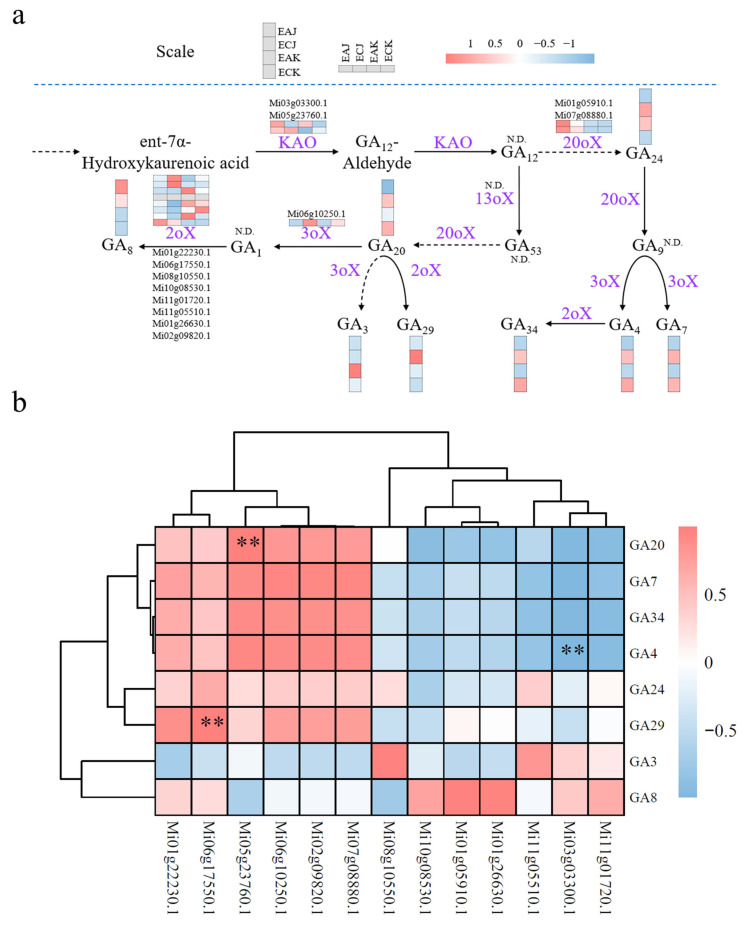
Expression of gibberellin biosynthetic enzymes and association with gibberellin levels in mango fruit (**a**) Proposed biosynthetic pathway of gibberellins and the relative expression of candidate genes across the pathway in mangoes. KAO, entkaurenoic acid oxidase; 20oX, GA 20-oxidase; 13oX, GA 13-oxidase; 3oX, GA 3-oxidase; 2oX, GA 2-oxidase; N.D., not detected. Pink indicates high expression; blue signifies low expression. (**b**) Correlation heatmap between the expression levels of genes involved in the gibberellin synthesis pathway and the levels of gibberellins. Pink indicates a positive correlation and blue denotes a negative correlation. Higher color intensity signifies a stronger correlation. Data were analyzed using Pearson correlation coefficient. Asterisks indicate significant correlations (** *p*<0.01).

Expression of plant-hormone signal transduction pathway genes and GA concentrations differed significantly between EA and EC fruits. Therefore, we analyzed the expression of GA biosynthetic genes and compared their transcript abundances with the measured GA concentrations in the two fruit types (Figure 5a). In total, 13 differentially expressed genes were identified including two *KAO*, two *GA20oX*, eight *GA2oX*, and one *GA3oX* genes (Table S4). Among the biosynthetic enzymes examined, ent-kaurenoic acid oxidase (*KAO*, *Mi05g23760.1*) stood out as the only gene that was both significantly differentially expressed between EA and EC fruits and showed the strongest association with the observed variation in GA_20_ (the biosynthetic precursor of GA_1_, GA_3_, GA_4_, and GA_7_) content (Figure 5b, Tables S5 and S6). Multiple sequence alignment and phylogenetic analysis showed that MiKAO genes was highly consistent with their homologs in *Arabidopsis* (Figure S4). Analysis of *KAO* gene expression in different tissues of the ‘Alphonso’ mango indicated high expression in roots and seeds, suggesting that these genes play important roles in stress responses and in the regulation of fruit hormones (Figure S5). These results nominate KAO as a prime candidate mediating the divergent GA profiles in EA versus EC fruit, and it warrants further functional validation to determine its causal role in regulating fruit-specific GA biosynthesis.

## 4. Discussion

Embryo-absent (EA) mangoes are preferred in food processing because they yield a higher proportion of edible tissue—up to 95% of total fruit mass—thereby reducing manual labor for seed removal. EA fruits also often contain higher concentrations of flavor compounds and soluble sugars, which increase perceived sweetness. For example, EA ‘Keitt’ mangoes exhibited 1.2-fold higher soluble solids than embryo-containing (EC) fruit (Figure S1), making them particularly suitable for production of dried mango, mango ice cream, and mango juice. Similar trends have been observed in other taxa: sensory evaluations of jams prepared from wild Physalis indicate that products derived from seeded varieties receive lower consumer acceptance than those from seedless varieties [23]. Reduced fruit growth can increase concentrations of soluble solids and flavor compounds by decreasing dilution and redirecting carbon away from structural growth into primary and secondary metabolic pathways. Although EA fruits are highly demanded, their development heavily depends on the extensive use of fruit development regulators. Our study found that EA and EC mangoes are morphologically similar at 30 DAB but diverge by 45 DAB, with EA fruits—particularly the ‘Keitt’ cultivar—showing markedly reduced vertical and horizontal growth (Figure 1a), consistent with a previous study implicating that the embryo in regulating cell division and expansion [14]. The developing embryo functions as a strong metabolic sink and as a source of growth-promoting hormones (e.g., auxin, cytokinins, gibberellins) that stimulate cell division and expansion. Consequently, embryo absence diminishes sink strength and limits fruit growth by restricting cell proliferation and expansion. These results define the 30–45 DAB period as the critical developmental window when targeted hormonal regulation is needed to correct developmental delays in EA mango fruits and ensure optimal growth and quality for high-value processing.

Fruit development is a prolonged process accompanied by changes in various metabolites; hormones, particularly gibberellins (GAs), play key roles in regulating fruit shape and growth. Our GA-targeted metabolomic analysis indicates that deficiencies in GA_4_ and GA_7_ impair the development of EA mangoes (Figure 2c), resulting in malformed or otherwise developmentally compromised fruits. Furthermore, exogenous application of GAs significantly promoted fruit development in EA ‘Jinhuang’ mango, with GA_4_+_7_ being more effective than GA_3_ (Figure 3b). These findings suggest that endogenous GA_4_ and GA_7_ are crucial drivers of elongation growth in EA mango fruits, offering promising strategies for improving fruit size and quality through targeted hormone treatments. These results align with previous reports in pear [24] and grape [25], where GAs play a key role in fruit set and subsequent development. Seedless fruit development observed in DELLA mutants is directly attributable to constitutive activation of GA signaling, and auxin-induced parthenocarpy operates entirely via GA signaling in Arabidopsis [26]. GAs are the main drivers of fruit enlargement, promoting cell expansion via GID1–DELLA signaling and activation of expansions [14]. In practice, GA is central to size management and is often applied during early fruit development, frequently together with the synthetic cytokinin CPPU, which promotes cell division and sink strength [27]. CPPU is used at low rates (≤5 ppm) to avoid negative effects on maturation and postharvest quality, and co-application with GA can yield additive or synergistic increases in cell number and size. Applied together, CPPU and GAs can increase both cell number and cell size, producing additive or synergistic effects on fruit growth. Given CPPU’s widespread use to regulate fruit size in crops such as kiwifruit and melon [28]^,^ [29], its physiological and molecular effects on mango fruit development merit systematic investigation.

Complementing these biochemical findings, transcriptome analysis identified 1476 differentially expressed genes (DEGs) enriched in hormone signal transduction and secondary metabolism pathways, underscoring the central role of phytohormones in fruit morphogenesis. This regulatory network controls two fundamental growth processes: cell division—primarily regulated by auxin signaling—and cell expansion, whose regulatory mechanisms are less well characterized [30]. Evidence indicates that auxin and GAs synergistically promote cell expansion; exogenous applications of both phytohormones can induce the initiation of fruit set and development in the absence of fertilization [11]^,^ [31]. Among these DEGs, *ent-kaurenoic acid oxidase* (*KAO*)—a key enzyme catalyzing critical steps in GA biosynthesis—exhibited reduced expression in EA fruits, strongly correlating with diminished GA_20_ levels (Figure 5b). This relationship underscores the essential regulatory role of *KAO* in GA biosynthesis and fruit development, consistent with previous studies demonstrating KAO’s influence on plant development in other species. For example, a single recessive G to A mutation in *CsKAO* in the cucumber mutant introduces a premature stop codon, reducing GA biosynthesis and endogenous GA levels, perturbing auxin distribution and cell elongation, and causing dwarfism that is rescued by application of exogenous GA_3_ [32]. Collectively, these results provide a comprehensive understanding of the hormonal and molecular mechanisms underpinning fruit elongation in EA mangoes and underscore the potential of manipulating specific GA biosynthetic pathways and isoforms to enhance fruit development and commercial value.

In summary, our results indicate that the deficiency of GA_4_ and GA_7_, resulting from the downregulation of GA biosynthetic genes such as KAO, explains the impaired growth observed in EA mango fruits. The superior efficacy of GA_4+7_ treatments compared to GA_3_ highlights the critical role of specific GA isoforms in regulating fruit development. Future studies should aim to functionally validate candidate genes, including KAO, and investigate the interactions among different hormonal pathways to fully elucidate the molecular mechanisms governing EA fruit development. These findings provide a solid molecular foundation to inform agronomic strategies for enhancing the yield and quality of EA mango cultivars, presenting promising opportunities for commercial production.

## 5. Conclusions

Our comparative morphological, hormonal, and transcriptomic analyses identify the 30–45 days after bloom (DAB) window as a critical phase during which developmental divergence between embryo-containing (EC) and embryo-absent (EA) mango fruits begins. Morphological differences appear by 45 DAB, with EA fruits showing reduced vertical and horizontal growth and altered shape index, accompanied by higher soluble solids in at least one cultivar. Targeted GA metabolomics at 30 DAB revealed a consistent deficiency of GA_4_ and GA_7_ in EA fruits of both ‘Keitt’ and ‘Jinbaihua’, pinpointing these bioactive GAs as likely limiting factors for EA fruit growth. Exogenous application experiments further demonstrated that combined GA_4+7_ treatment more effectively promoted elongation and overall development of EA fruits than the commonly used GA_3_. Transcriptome profiling showed extensive reprogramming of hormone signaling and secondary metabolite pathways between EA and EC fruits, and investigation of GA biosynthetic genes revealed ent-kaurenoic acid oxidase (KAO) as the strongest transcript correlate of altered GA precursor levels. Together, these results implicate a deficiency in GA_4_/GA_7_—potentially driven by differential KAO expression—in the arrested growth of EA mango fruits and suggest that targeted manipulation of GA_4/7_ levels or KAO activity represents a promising approach to restoring normal seedless fruit development. Future work should pursue functional validation of KAO, map the spatiotemporal dynamics of GA biosynthesis in seed versus pericarp, and optimize GA_4+7_ application regimes for practical use in mango production.

## Data Availability

The original contributions presented in the study are included in the article/Supplementary Materials, further inquiries can be directed to the corresponding authors.

## Supplementary Materials

The following supporting information can be downloaded at: https://www.mdpi.com/article/10.3390/foods14213705/s1, Figure S1. Soluble solids of ‘Keitt’ and ‘Jinbaihua’ mature fruits. Data were examined using two-way ANOVA, with asterisks indicating significant differences (** *p* < 0.01). EA, embryo-absent mature fruit; EC, embryo-containing mature fruit. Figure S2. Volcano plot of differentially expressed genes. Volcano plot of differential gene expression between embryo-absent and embryo-containing ‘Keitt’ (a) and ‘Jinbaihua’ (b) fruits. The x-axis shows the fold change in gene expression, and the y-axis shows the significance level of differential expression. Red dots indicate upregulated differentially expressed genes, blue dots indicate downregulated differentially expressed genes, and gray dots indicate genes that are not differentially expressed. Figure S3. Sample variability and differential gene statistics. (a) Sample PCA plot. (b) Differential gene statistics plot. The x axis shows the comparison groups, and the y axis indicates the numbers of upregulated, downregulated, and total differential genes. EAK, embryo-absent ‘Keitt’ fruit; ECK, embryo-containing ‘Keitt’ fruit; EAJ, embryo-absent ‘Jinbaihua’ fruit; ECJ, embryo-containing ‘Jinbaihua’ fruit. Figure S4. Evolutionary analysis of KAOs. Evolutionary tree and multiple sequence alignment of mango KAOs and some of arabidopsis. Phylogenetic analysis and the alignment were at the protein level. Numbers above branch lines represent bootstrap values. The scale bar represents sequence divergence. Figure S5. Heatmap of mango KAO gene expression in the ‘Alphonso’ transcriptome across various tissues. Red and blue denote high and low gene transcription levels, respectively. Table S1. Parent and daughter ions and fragmentation energy for each GAs. Table S2. Table of gibberellin detection results. Table S3. List of differentially expressed genes. Table S4. Table of gibberellin biosynthesis gene count value. Table S5. Correlation coefficients of gibberellin with genes involved in its metabolism. Table S6. *p*-values for the correlations between gibberellin levels and genes involved in its metabolism.

## Abbreviations

The following abbreviations are used in this manuscript:

GA	Gibberellin
DAB	Days after blooming
EA	Embryo-absent
EC	Embryo-containing
