# Supplementary material for: GA4/GA7 Deficiency and Downregulated Ent-Kaurenoic Acid Oxidase Impair Seedless Mango Fruit Development
Source: Foods. 2025 Oct 30;14(21):3705. doi: 10.3390/foods14213705 (PMC12610870; doi:10.3390/foods14213705)
Supplement: Supplementary file 1 [file foods-14-03705-s001.zip › Supplementary data.pdf]

Supporting data

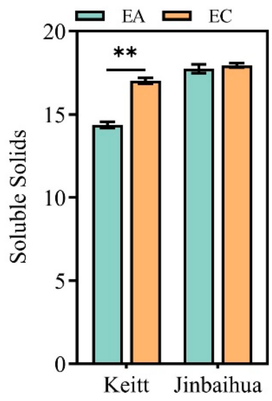

Figure S1. Soluble solids of ‘Keitt’ and ‘Jinbaihua’ mature fruits. Data were examined using two-way ANOVA, with asterisks indicating significant differences (**$p < 0.01$). EA, embryo-absent mature fruit; EC, embryo-containing mature fruit.

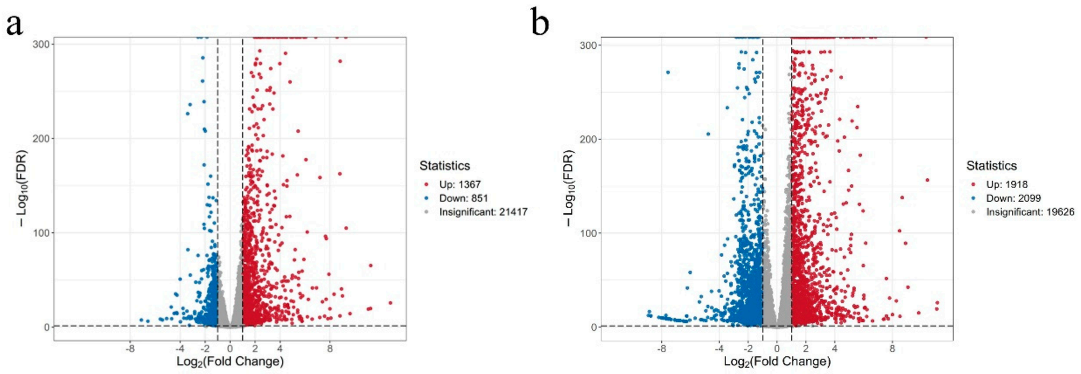

Supplementary Figure S2. Volcano plot of differentially expressed genes. Volcano plot of differential gene expression between embryo-absent and embryo-containing ‘Keitt’ (a) and ‘Jinbaihua’ (b) fruits. The x-axis shows the fold change in gene expression, and the y-axis shows the significance level of differential expression. Red dots indicate upregulated differentially expressed genes, blue dots indicate downregulated differentially expressed genes, and gray dots indicate genes that are not differentially expressed.

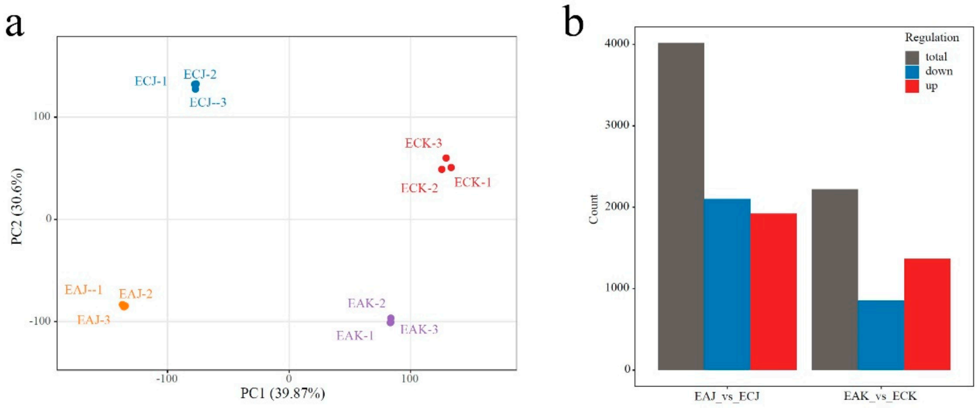

Supplementary Figure S3. Sample variability and differential gene statistics. (a) Sample PCA plot. (b) Differential gene statistics plot. The x-axis shows the comparison groups, and the y-axis indicates the numbers of upregulated, downregulated, and total differential genes. EAK, embryo-absent ‘Keitt’ fruit; ECK, embryo-containing ‘Keitt’ fruit; EAJ, embryo-absent ‘Jinbaihua’ fruit; ECJ, embryo-containing ‘Jinbaihua’ fruit.

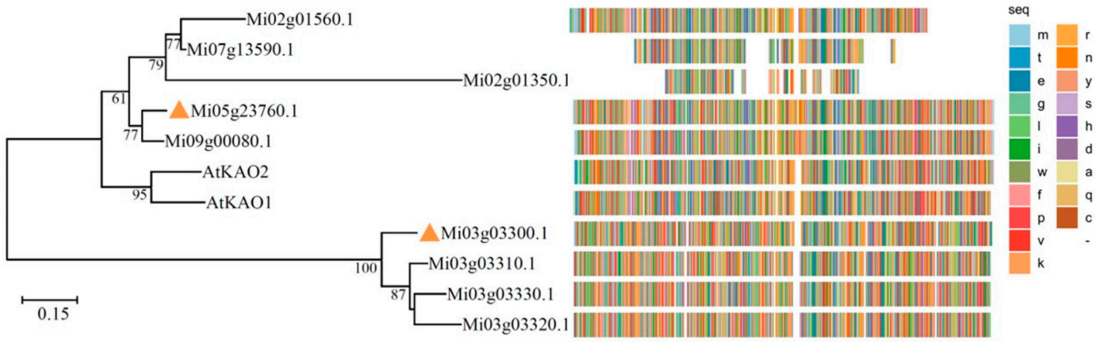

Supplementary Figure S4. Evolutionary analysis of KAOs. Evolutionary tree and multiple sequence alignment of mango KAOs and some of arabidopsis. Phylogenetic analysis and the alignment were at the protein level. Numbers above branch lines represent bootstrap values. The scale bar represents sequence divergence.

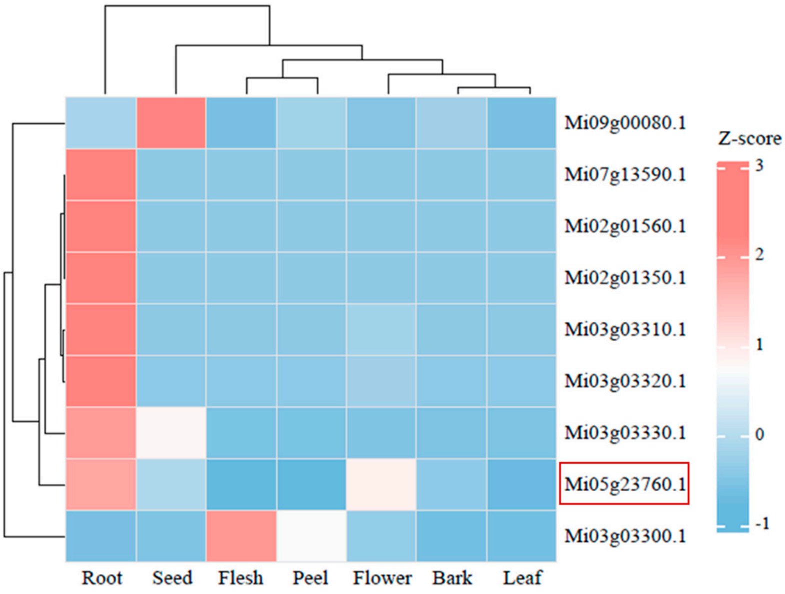

Supplementary Figure S5. Heatmap of mango KAO gene expression in the ‘Alphonso’ transcriptome across various tissues. Pink and blue denote high and low gene transcription levels, respectively.
